# The effects of self‐efficacy training on associative memory and brain function in older adults with mild cognitive impairment

**DOI:** 10.1002/alz.091936

**Published:** 2025-01-09

**Authors:** Raphael Gabiazon, Samantha Marshall, Amy Swayze, James Van Riesen, Gianna Jeyarajan, Jennifer Hanna Al‐Shaikh, Lindsay S Nagamatsu

**Affiliations:** ^1^ Western University, London, ON Canada

## Abstract

**Background:**

Older adults with mild cognitive impairment (MCI) are known to have declines in cognition greater than that expected for an individual’s age and education level but does not significantly interfere with daily functioning. Memory impairments during the early stages of MCI may predict conversion to Alzheimer’s disease (AD). This impairment can be especially prominent in associative memory – the ability to learn and remember the relationship between two unrelated items. While age‐related memory impairments are often attributed to biological and physiological changes, psychosocial factors could also play a role. A notable psychosocial factor that may affect memory is self‐efficacy – an individual’s confidence in their ability to carry out actions to meet situational or domain‐specific demands. Despite previous research showing a relationship between enhanced self‐efficacy and improved memory performance in older adults, the neural correlates that may explain this relationship remain unclear. Therefore, we developed a six‐week self‐efficacy training intervention to examine its effects on memory and corresponding changes in functional brain activation among older adults with MCI.

**Method:**

Twenty‐eight participants were randomized and allocated into either a memory self‐efficacy intervention (n = 14) or general education control group (n = 14). Over six weekly 1.5‐hour group sessions, participants attended either classes that focused on building memory self‐efficacy or general education covering factual and world knowledge. At baseline and endpoint measurements, memory performance and functional brain activation were assessed using an associative memory task during a functional magnetic resonance imaging scan, while memory self‐efficacy was measured with the Multifactorial Memory Questionnaire. Post‐intervention group differences for each outcome measure were analyzed using multiple linear regression models.

**Result:**

We found changes in functional activation patterns during the associative memory task in the memory self‐efficacy training group as a result of the intervention. Specifically, key regions in the brain that are critical for associative memory performance were implicated, suggesting that memory self‐efficacy training can change the way we process and remember information.

**Conclusion:**

This study provides insight into how psychosocial factors like self‐efficacy may influence age‐related memory impairments and AD progression, demonstrating self‐efficacy training's potential to enhance memory in at‐risk populations.

